# Hemodynamic numerical simulations of the disturbance due to intracoronary flow measurements by a Doppler guide wire

**DOI:** 10.1186/s12938-016-0234-6

**Published:** 2016-10-10

**Authors:** Kamil J. Chodzyński, Simone Gremmo, Omer F. Eker, Jacques Lalmand, Adel Aminian, Daniel Ribeiro de Sousa, Karim Zouaoui Boudjeltia, Grégory Coussement

**Affiliations:** 1 Laboratoire de Médecine Expérimentale (ULB 222 Unit), Route de Gozée 706, 6110 Montigny-le-Tilleul, Belgium; 2 Faculté Polytechnique de Mons-Service Fluides-Machines, Université de Mons, 53, rue du Joncquois, 7000 Mons, Belgium; 3 Service of Cardiology, CHU Charleroi, Boulevard Zoé Drion, 16000 Charleroi, Belgium; 4 Service de Neuroradiologie Hôpital Gui de Chauliac, 80 Avenue Augustin Flich, 34295 Montpellier cedex 05, France

**Keywords:** Doppler guide wire, Numerical simulations, Flow disturbance, Unsteady pulsatile inlet boundary condition

## Abstract

**Background:**

Since hemodynamics plays a key role in the development and evolution of cardiovascular pathologies, physician’s decision must be based on proper monitoring of relevant physiological flow quantities.

**Methods:**

A numerical analysis of the error introduced by an intravascular Doppler guide wire on the peak velocity measurements has been carried out. The effect of probe misalignment (±10°) with respect to the vessel axis was investigated. Numerical simulations were performed on a realistic 3D geometry, reconstructed from coronary angiography images. Furthermore, instead of using Poiseuille or Womersley approximations, the unsteady pulsatile inlet boundary condition has been calculated from experimental peak-velocity measurements inside the vessel through a new approach based on an iterative Newton’s algorithm.

**Results:**

The results show that the presence of the guide modifies significantly both the maximum velocity and the peak position in the section plane; the difference is between 6 and 17% of the maximum measured velocity depending on the distance from the probe tip and the instantaneous vessel flow rate. Furthermore, a misalignment of the probe may lead to a wrong estimation of the peak velocity with an error up to 10% depending on the probe orientation angle.

**Conclusions:**

The Doppler probe does affect the maximum velocity and its position during intravascular Doppler measurements. Moreover, the Doppler-probe-wire sampling volume at 5.2 and 10 mm far from the probe tip is not sufficient to prevent its influence on the measurement. This should be taken into account in clinical practice by physicians during intravascular Doppler quantification. The new numerical approach used in this work could potentially be helpful in future numerical simulations to set plausible inlet boundary conditions.

**Electronic supplementary material:**

The online version of this article (doi:10.1186/s12938-016-0234-6) contains supplementary material, which is available to authorized users.

## Background

Hemodynamics plays an important role in cardiovascular diseases and their treatments. Physician’s decisions about surgical intervention are mostly based on information provided by monitoring physiological flow parameters such as blood velocity and pressure. For instance, the decision to deploy a stent in a stenotic artery is based on the pressure measured upstream and downstream the lesion. The accuracy of such specific information is essential for improving the surgery outcomes and reducing the risks. In the field of hemodynamics, a distinction can be made between non-invasive measurement techniques (e.g. transthoracic ultrasound probe and magnetic resonance imaging [1]) and invasive techniques (e.g. intravascular coronary catheter probes [2–4]). Even if the first ones do not influence the parameters to be measured and should be preferred to the latter, they cannot be used under all circumstances (i.e. non reachable target vessel) and they present the drawback of not allowing pressure measurement. The main problem with the invasive techniques relies on the perturbations induced by the measurement devices that may lead to measurement errors and eventually to wrong medical decisions.

In the literature, several experimental [2, 4] and numerical [5, 9] studies describe approaches to assess the disturbance on the flow caused by a catheter. These studies have however some methodological limitations. The experimental investigation made by Doucette et al. [4] proposes a comparison between mean flow velocity measurements, carried out with an electromagnetic flow meter, and a Doppler probe, for straight and tortuous tube models in steady and unsteady conditions. The comparison, assuming a time-averaged parabolic velocity profile, shows a good result correlation for low flow regimes, however, some hardly explainable discrepancies are obtained for high flow rates when using a tortuous tube model.

The numerical investigations about the influence of a catheter were carried out using straight pipes with stenosis [5, 7] and/or a curved pipe model [6] excluding more complicated geometries (e.g. real vessels with a bifurcation). The blood flow models are based on steady or pulsatile laminar incompressible Newtonian flow with a fully developed parabolic flow profile as inlet or outlet boundary conditions [8, 9]. In [6, 7], the effect of the relative size of the catheter on the flow is estimated around 5–10% for the pressure and 15–21% for average velocity. The variability depends mostly on the size of the catheter with respect to the vessel size.

In order to improve the physician’s decision process, a better understanding of the flow disturbance induced by a catheter and its positioning with respect to the vessel axis and flow direction is needed. Catheter insertion may locally change blood flow properties and physicians should be aware of that impact when they take samples for biological manipulations.

The aim of this work is to assess, through CFD simulations, the perturbations introduced by the intravascular catheter probe on the flow velocity in a realistic 3D bifurcated vessel geometry. This preliminary study aims at assessing the reliability of experimental results used for comparison with numerical simulations. A new approach has been used to impose the pulsatile inlet boundary condition; instead of using the classic fully developed parabolic profile or the Womersley solution [10], the periodic unsteady inlet flow has been calculated using a Newton’s iterative method to satisfy the measured unsteady velocity inside the vessel. Furthermore, a sensitivity analysis of the catheter probe position inside the vessel on the velocity measurements is included.

## Methods

### In vivo instantaneous velocity measurements

Measurements were made using a ComboMap^®^ analysis unit (Volcano Corp.) that includes: a Doppler flow intravascular wire (ComboWire^®^, Volcano Corp.) to measure the blood instantaneous velocity in a coronary artery. The ComboWire^®^ is an intravascular wire probe, whose diameter is 0.36 mm and the flow sensor is located on its tip [11].

Special care was taken to keep the wire tip at the centre of the artery lumen for optimal measurements. The patient was selected among those already undergoing a coronarography and the measurements were performed in a coronary artery devoid of any pathology. As imposed by the current daily practice, the diagnostic procedure was carried out with a 2D scanner. The patient had normal left ventricular systolic function and the ComboWire^®^ was placed in the proximal part of the left anterior descending artery (LAD).

**Fig. 1:**
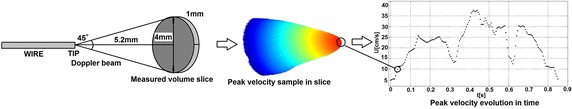
Doppler guide wire—acquisition of peak velocity. Position of the measured volume slice with respect to the tip guide wire (*left*) and peak evolution in time reconstruction (*right*) from the measured samples (*centre*)

The methodology for the in vivo velocity measurement is presented in Fig. 1. The sensor sends a Doppler beam with 45° insonation angle and the volume slice (cylinder of 4 mm diameter and 1 mm height) located at 5.2 mm ahead of the sensor tip. From the recorded samples, the peak velocity in that volume slice was extracted and this procedure was repeated every 5 ms in order to obtain the time evolution of the velocity signal.

**Fig. 2:**
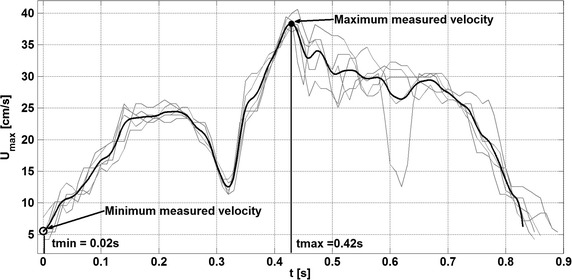
Measured coronary velocity: raw data and post-processed data. A real coronary artery signal measured in a patient indicates a significant variability from cycle to cycle (length of one cycle equals 0.83 s). The figure depicts the in vivo signal, divided in periods of the same length (*thin gray lines*) and the resulting averaged filtered velocity profile (*thick line*). In addition, minimum and maximum measured velocities at a given time are marked as *circles*

The in vivo experimental measurements in the coronary artery were recorded and then submitted to filtering procedures. Firstly, a post-processing was applied to remove artefacts possibly caused by movements of the patient and/or of the sensor [12]. Secondly, to obtain a representative periodic measurement signal, an average of all the filtered cycles was constructed (represented in Fig. 2 as the solid black line). Lastly, the resulting averaged unsteady pulsatile artery velocity signal was decomposed into the Fourier series for further analysis; 50 harmonics were used to obtain good accuracy in the Fourier representation of the unsteady periodic pulsatile velocity [13].

### Extracting 3D vessels geometry from 2D angiography image

**Fig. 3:**
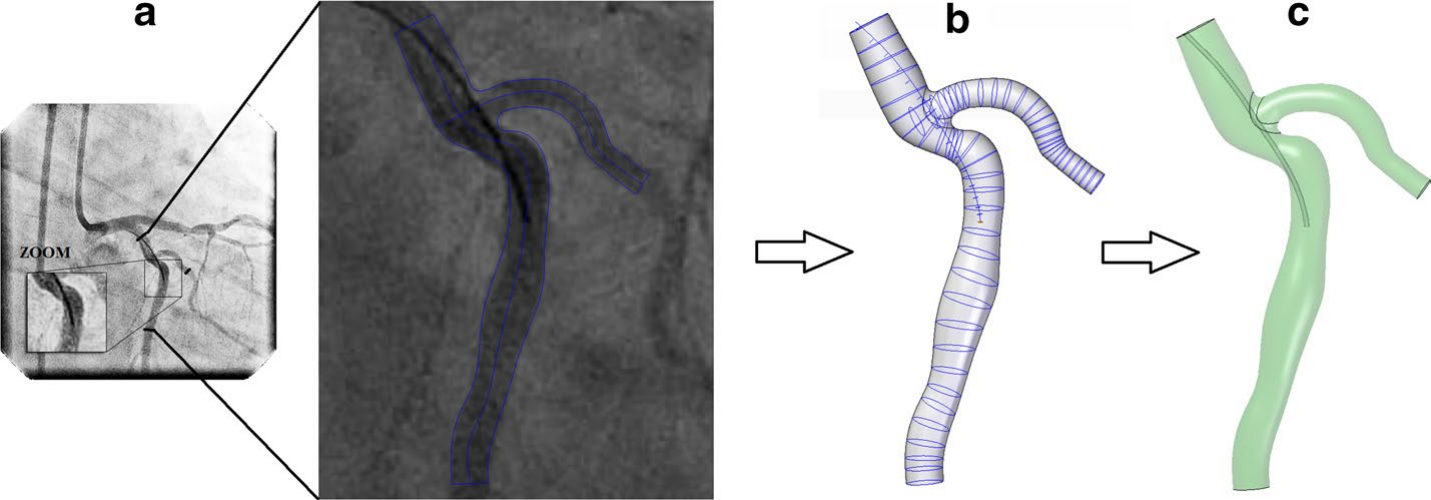
From angiographies images to CAD model for CFD computation. **a**, *left* The quantitative coronary angiography (QCA) images performed during the measurements, (**a**, *right*) outline the 2D geometry, **b** 3D skeleton definition by contour revolution and **c** closed volume generation

The vessel profile was extracted from the quantitative coronary angiography (QCA) images performed during the measurements (Fig. 3a), and the 3D geometry of the vessel was reconstructed using CAD software. The manual geometry modelling procedure includes three steps:

1. The extraction of wall boundaries of the vessel and the probe wire based on the angiography recordings (see image Fig. 3a).
2. The manual segmentation of the vessel and the wire by inserting cross section between boundaries limits (see Fig. 3b).
3. The construction of a protrusion by extruding a cross section along a defined path (the centre of the vessel, Fig. 3c). Finally, the geometry was scaled to fit real dimensions and exported to STAR-CCM + v 9.02 as a triangulated surface (.stl file format) to perform CFD simulations.

### CFD settings and implementation

In order to assess the impact of the probe wire in the vessel lumen on the flow and on the measurement error, two configurations were compared: “with wire” and “without wire”. The comparison is based on the results obtained by CFD flow simulations and is given both in terms of pressure and velocity fields. For these two geometries the flow solutions were obtained using the finite volume flow solver of STAR-CCM + v 9.02. Subsequently, the models were meshed using full hexahedral meshes and to resolve the thin boundary layer, three prismatic layers were added in a direction perpendicular to the wall. Moreover, instead of a classical global mesh refinement convergence study, the mesh cell-size distribution was set in a more effective way, using a local mesh refinement based on the velocity field. A criterion to assess the solution quality [14–16] is to limit the local Reynolds Number by imposing in this study ${\text{Re}}_{\text{local}} = \frac{{{\text{u}}_{\text{local}} \sqrt[3]{{{\text{volume}}_{\text{cell}} }}}}{{\upsilon_{\text{local}} }} <10$ everywhere. The maximum local Reynolds equal to 10 turned out to be the best compromise between our computational resources (time and memory) and solution accuracy.

The number of cells is 3.2 million for the model without wire and 3.1 million for the model with wire. The difference is due to the presence of the wire which is represented as a void in the geometry and that globally decreased the number of cells, despite the additional boundary layer refinement along the wire walls.

For these unsteady pulsatile simulations, laminar non-Newtonian incompressible flow behaviour in a rigid vessel was assumed. Using the patient’s maximum velocity and the vessel diameter, the maximal Reynolds number was ${\text{Re}}_{ {\rm max} } = \frac{{{\text{u}}_{ {max} } {\text{D}}}}{\upsilon } = 260$ validating the assumption of laminar flow in the vessel. The blood has a small compressibility that can be neglected, in particular when studying local aspects of flow and a constant density ρ = 1055 kg/m^3^ was therefore imposed. In normal conditions, blood is a heterogeneous media principally composed of about 55% liquid plasma and 45% red blood cells (RBCs) in suspension in the plasma. The concentration of RBCs modifies the apparent viscosity of blood. Moreover, the apparent viscosity changes depending on the shear rate, leading to the non-Newtonian behaviour of the blood, where the viscosity decreases as shear rate increases. During the intervention the hematocrit (Hct) level was 36.6% and the Carreau equation was used to model the blood flow non-Newtonian behaviour. This model (Eq. 1) is based on four parameters that are expressed as a function of the hematocrit level (Hct) [13]:

1 $${\varvec{\upmu}}({\dot{\mathbf{\gamma }}}, {\mathbf{Hct}})\, = \,{\varvec{\upmu}}_{\infty }^{{\varvec{Hct}}} + \left( {{\varvec{\upmu}}_{0}^{{\varvec{Hct}}} - {\varvec{\upmu}}_{\infty }^{{\varvec{Hct}}} } \right)\left( {{\mathbf{1}} + \left( {{\varvec{\uplambda}}^{{\varvec{Hct}}} {\dot{\mathbf{\gamma }}}} \right)^{{\mathbf{2}}} } \right)^{{\frac{{{\mathbf{n}}^{{\varvec{Hct}}} - {\mathbf{1}}}}{{\mathbf{2}}}}}$$

The Carreau model parameters were based on the experimental data published in [13]: ${\upmu }_{0} = 2.17 \times 10^{- 2} \,{\text{Pa}}\,{\text{s}}$ for the viscosity at zero shear rate, ${\upmu }_{\infty} = 2.61 \times 10^{- 2} \, {\text{Pa}}\,{\text{s}}$ for the viscosity at infinite shear rate, λ = 1.48 s for the relaxation time parameter, n = 0.40 for the dimensionless coefficient. As the use of elastic pipes does not change significantly the results [13], the rigid-pipe approximation of the blood vessel was reasonably applied.

Additionally, a cylindrical extension was added to the vessel inflow and the two outflows in order to guarantee that the boundary conditions do not affect the flow in the region of interest. The length added to the vessel inflow and outflows was estimated using a semi-empirical law valid for steady laminar flow [17]:

2 $$\frac{{{\mathbf{L}}_{{\mathbf{e}}} }}{{\mathbf{D}}} \approx {\mathbf{0}}{\mathbf{.0575Re}}$$

Since, for incompressible flows, the pressure variations inside the rigid vessel remain the same regardless the absolute pressure value on the outlet, the atmospheric pressure is arbitrary imposed at the outlet boundary. On the inlet surface a velocity u(t) uniform in space is imposed. As mentioned, a new approach to calculate the inlet boundary condition was applied in order to fit the measured values inside the vessels (see description in “Boundary inlet conditions—Newton’s approach” section).

These CFD calculations are based on the incompressible unsteady Navier–Stokes equations of mass and momentum conservation:

3 $$\vec{\nabla } \cdot {\vec{\text{u}}} = 0\quad {\text{mass equation}}$$

4 $${\uprho }\frac{{\partial {\vec{\text{u}}}}}{{\partial {\text{t}}}} + {\uprho }\left( {{\vec{u}} \cdot \vec{\nabla }} \right){\vec{u}} = - \vec{\nabla }{\text{p}} + \vec{\nabla }\left( {{\upmu }\left( {\vec{\nabla }{\vec{\text{u}}} + \left( {\vec{\nabla }{\vec{\text{u}}}} \right)^{\text{T}} } \right)} \right) + {\uprho \vec{\text{f}}}\quad {\text{momentum equation}}$$

where, ${\vec{\text{f}}}$ is the external volume force, ${\upmu (\dot{\gamma )}}$ is dynamic viscosity that depends on shear rate tensor ${\dot{\upgamma }}$ for non-Newtonian fluids, with $\vec{\nabla }{\vec{u}}\, + \, (\vec{\nabla }{{\vec{u})}}^{\text{T}} = \, {\dot{\upgamma }}$.

Finally, to ensure the time accuracy of the simulation results, the time step for the time integration has to be wisely chosen. A convergence study was performed with three different time steps (1 × 10^−3^ s, 1 × 10^−4^ s, 1 × 10^−5^ s). Since no differences in the flow fields were quantifiable between the results obtained with a time step of 1 × 10^−4^ s and 1 × 10^−5^ s, the former was taken in order to minimize the global computational time. In addition, three periodic pulsatile cycles were necessary to ensure a periodic convergence of the simulation. For the sake of the analysis only the 3rd cycle is presented.

### Boundary inlet conditions—Newton’s approach

As the unsteady pulsatile flow conditions at the inlet of the computational domain are not directly available, it is necessary to indirectly evaluate them from the quantities measured inside the vessel. A steady Poiseuille flow or pulsatile Womersley’s solution is often used in this situation [10], however those methods are based on the assumption of straight pipe with constant section [18]. As our model is not a straight pipe and includes a bifurcation with changing sections, the following approach was applied to assess suitable inlet conditions.

By prescribing the outlet pressure, from the mass and momentum conservation laws, the inlet velocity unequivocally defines the velocity flow fields inside the vessel. Therefore, to impose pulsatile inlet conditions corresponding to the measurements inside the vessel, an iterative process was applied. The iterative search for the inlet condition can be seen as the minimization (e.g. lower than a given tolerance) of an error function quantifying the difference between measured and simulated velocity inside the volume slice. The Newton’s method has been implemented for the zero search:

5 $${\mathbf{u}}( {\mathbf{t}} )_{{\mathbf{i}} + 1} ={\mathbf{u}} ( {\mathbf{t}})_{\mathbf{i}} - \frac{{{\mathbf{e}} ( {{\hat{\mathbf{u}}}_{\text{i}} ( {\mathbf{t}} )})}}{{{\mathbf{e}}^{\prime} ( {{\hat{\mathbf{u}}}_{\mathbf{i}} ( {\mathbf{t}})})}}$$

where: u(t) is the velocity at the inlet, ${\text{e}}\left( {{\hat{\text{u}}}\left( {\text{t}} \right)} \right)$ is the error function and ${\text{e}}^{\prime} \left( {{\hat{\text{u}}}\left( {\text{t}} \right)} \right)$ is the first derivative of the error function. The error function is given by

6 $${\mathbf{e}}({\mathbf{t}})={{\hat{\mathbf{u}}}}_{{{\mathbf{sim}}{\mathbf{.}}}} ({\mathbf{t}}) - {{\hat{\mathbf{u}}}}_{{{\mathbf{mes}}{\mathbf{.}}}} ({\mathbf{t}})$$

where: ${\hat{\text{u}}}_{{{\text{sim}}.}} \left( {\text{t}} \right)$ is the peak velocity in the volume slice obtained by the CFD simulation, ${\hat{\text{u}}}_{{{\text{mes}}.}} \left( {\text{t}} \right)$ is the measured peak velocity in the volume slice using the Doppler guide wire.

To estimate the first derivative of the error e′, a one-sided finite difference was used:

7 $${\text{e}}^{\prime} ( {{\text{u}} ( {\text{t}} )} ) = \frac{{{\text{e}}\left( {{\text{u}}\left( {\text{t}} \right) + {\text{ du}}} \right) - {\text{e(u}}\left( {\text{t}} \right) )}}{\text{du}}$$

**Fig. 4:**
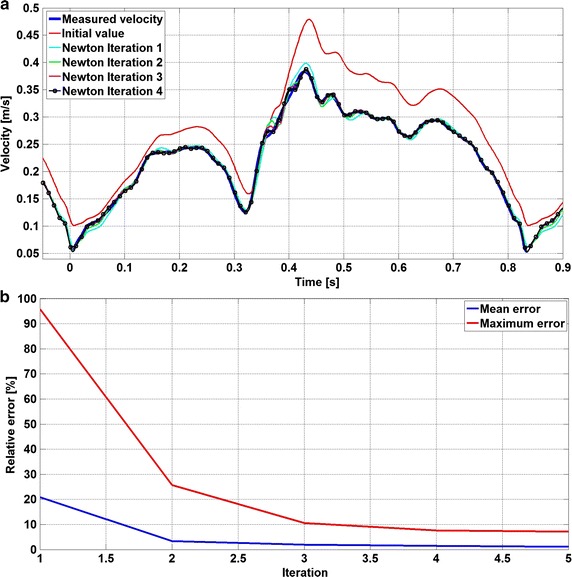
Peak velocity evolution in measuring volume slice during Newton’s iterations. **a** The Newton’s iterations of the peak velocity compared with in vivo measured velocity (*blue line*) inside the vessel are given. **b** The maximum and mean relative error values of error function e(u(t)) at each iteration to prescribe fitted inlet boundary condition are plotted

The evaluation of e′ requires to run an additional simulation whose inlet velocity is uniformly shifted by du = 0.01. After several tests, this value of du has been found to be a compromise between maximum and minimum velocity variation. Using a too high value for du would lead to a poor evaluation of the first derivative, especially for the smallest velocities, while with too small value of du the simulation would not show any significant difference for the highest velocities. Therefor for each Newton’s iteration a pair of simulations needed to be run to evaluate e′ with a reduction by a factor 4 of the maximum and mean errors after the first iteration (Fig. 4a). The Newton’s method is simple and its convergence rate is sufficiently fast as, in the present study, the initial guess was already quite good. Among the tested methods, the Newton’s was retained for its implementation simplicity and fast convergence rate. The Newton’s method stop criterion is based both on the maximum and average values of the error function:

- Maximum error = max(e(t)) < 5%
- Mean Error = mean(e(t)) < 1%

The CFD peak velocity curves for each Newton’s iteration are provided in (Fig. 4a). Except from the initial guess (red line), there is already a good overlap after the first iteration and the final iteration is satisfactorily superposed to the target measured velocity curve. In addition, the maximum and the mean values of the error function e(t) for each iteration are shown in (Fig. 4b): from initial guess set equal to the measured velocity, only 4 iterations are necessary to reach the thresholds for both the maximum and mean relative error.

**Fig. 5:**
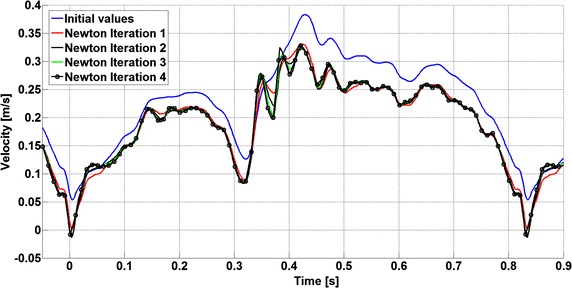
Inlet velocity condition during the Newton’s iterations. Each *coloured line* represents the time dependent inlet velocity for each Newton’s iteration (*blue line* is the first guess—*black* with the markers is the final solution)

A Matlab script was implemented in order to automatize the search for the unsteady inlet condition. This algorithm has 4 main steps: (1) Set the measured peak velocity inside the vessel as initial guess for the inlet velocity in the CFD solver; (2) Launch a pair of STAR-CCM + simulations, with inlet velocity u(t) and u(t) + du respectively; (3) Extract the CFD peak velocity and calculate the error function e(t); (4) Check whether the thresholds are respected or not, in which case update the inlet velocity using Eq. 5 and restart from point (2). The whole process was run on a computer having an Intel^®^ Core™ i7-2670QM processor with 16 GB of ram. In order to compute each iteration, it took about 22 h and to complete the whole process 17 days were needed. The inlet velocity boundary condition is plotted in Fig. 5. It can be noticed that the initial guess (blue line) overestimates the final values. Moreover, the convergence rate is slower where the highest frequencies are observed, due to fast acceleration or deceleration in the signal. Finally, the same calculated inlet velocity was set for the two configurations (e.g. with and without wire).

## Results

**Fig. 6:**
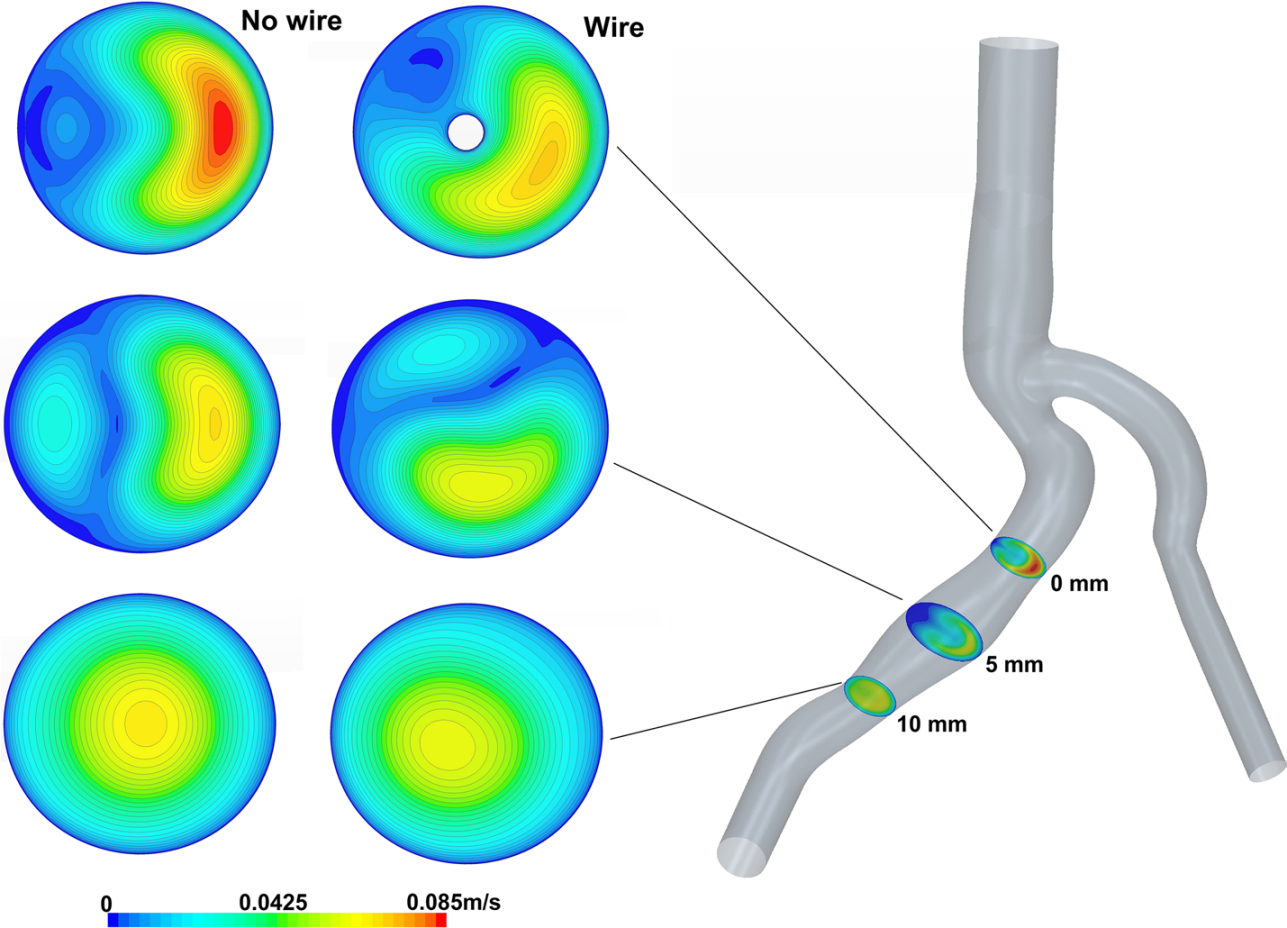
Flow field comparison at t = 0.02 s for 3 cutting planes. The figure presents the 2D velocity magnitude fields for the minimum velocity at t = 0.02 s. The 3 cutting planes are respectively 0, 5 and 10 mm far from the guide wire tip. To emphasize the guide wire influence, a comparison of the fields without the guide wire (*left*) and with (*right*) is also provided

**Fig. 7:**
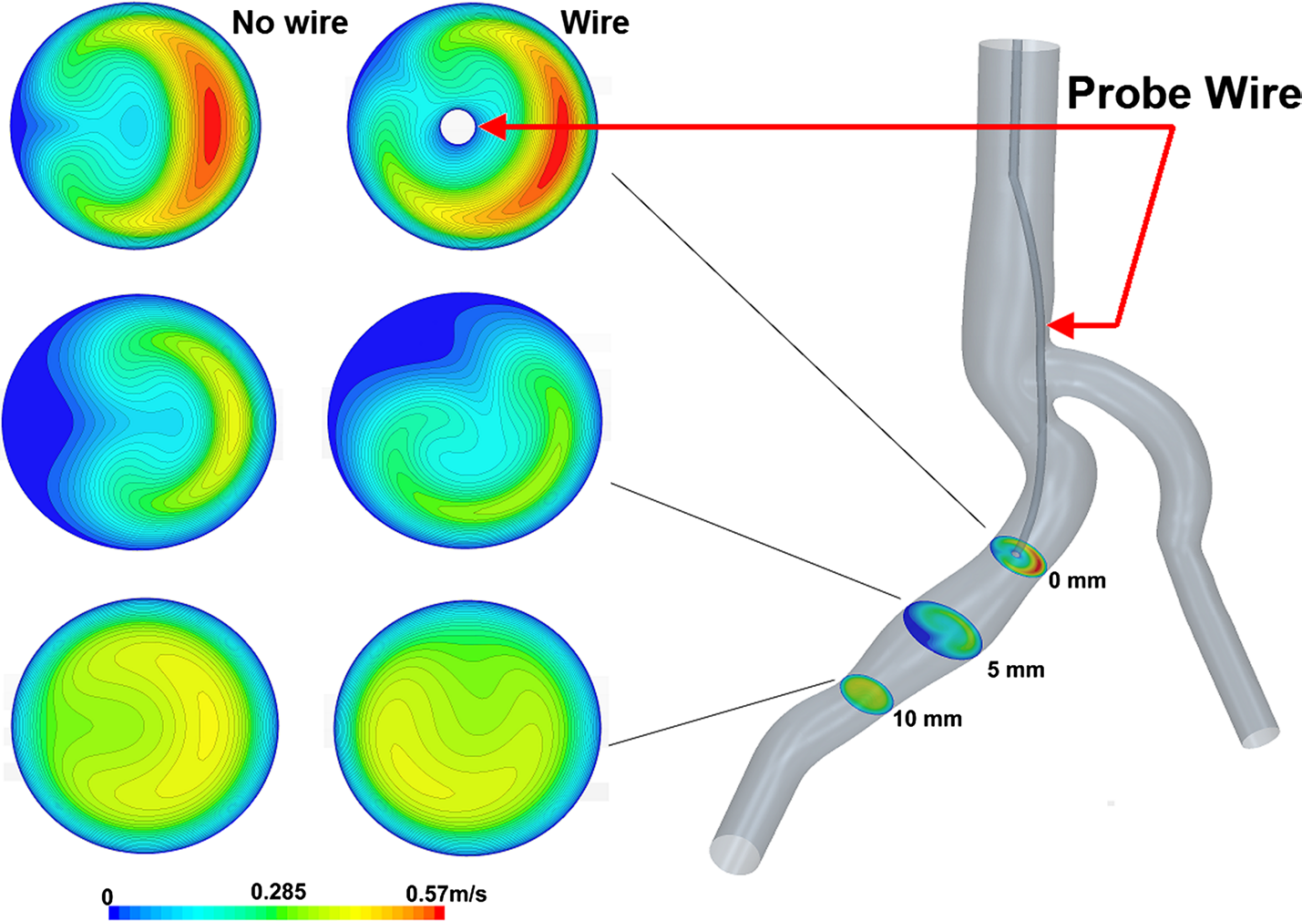
Flow field comparison at t = 0.44 s for 3 cutting planes. The figure presents the 2D velocity magnitude fields for the maximum velocity at t = 0.44 s. The cutting planes are respectively 0, 5 and 10 mm far from the guide wire tip. To emphasize the guide wire influence, a comparison of the fields without the guide wire (*left*) and with (*right*) is also provided

**Fig. 8:**
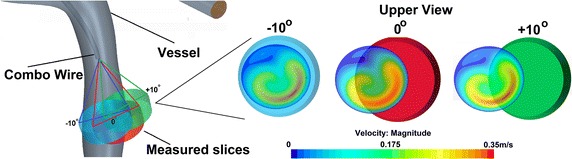
Investigated volume slices for different orientations. The figure presents the investigated volume slices and the covered regions in the vessels for three different angles (−10°—*blue cylinder*, 0°—*red cylinder*, +10°—*green cylinder*)

**Fig. 9:**
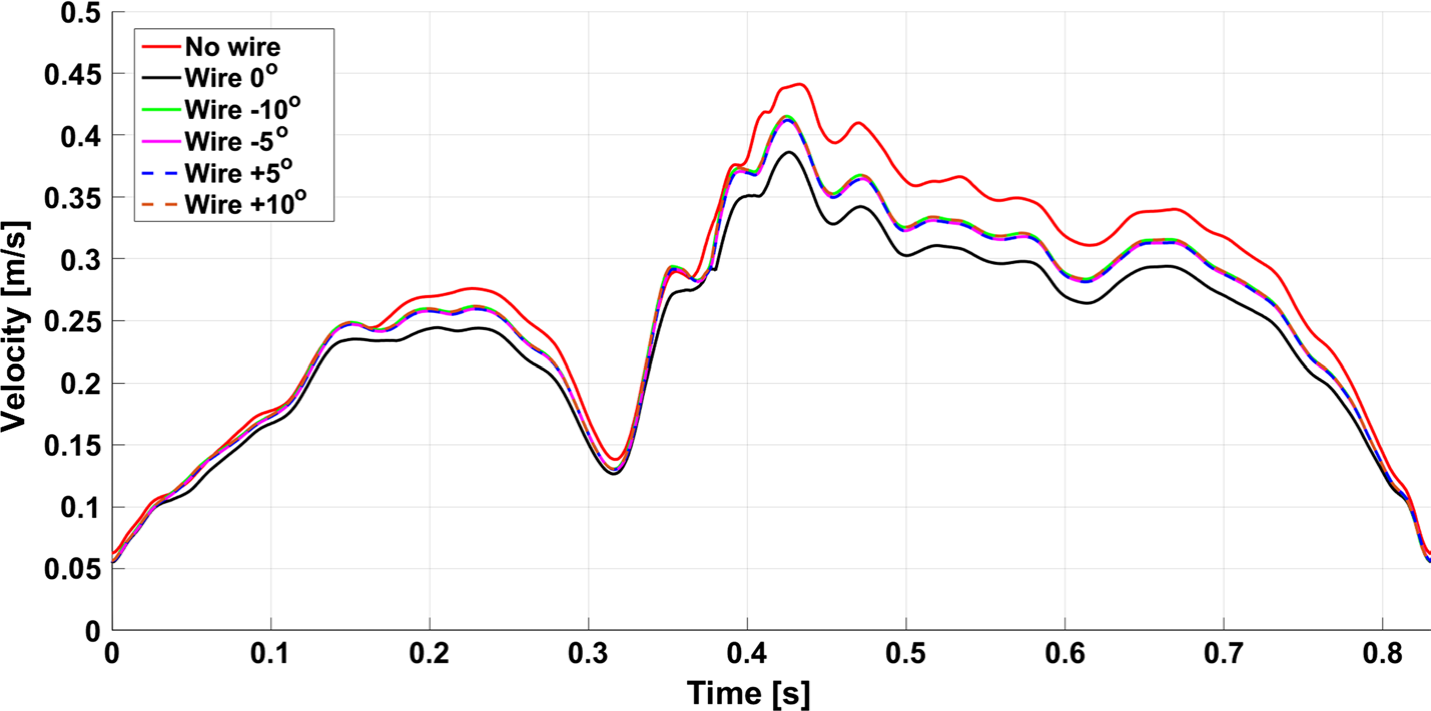
Analysis of the catheter probe position angles inside the vessel. The *figure* presents the maximum velocity evolution measured for different position of the catheter angles between ∓10°. In addition, for comparison a result for the case without the wire is also added

**Fig. 10:**
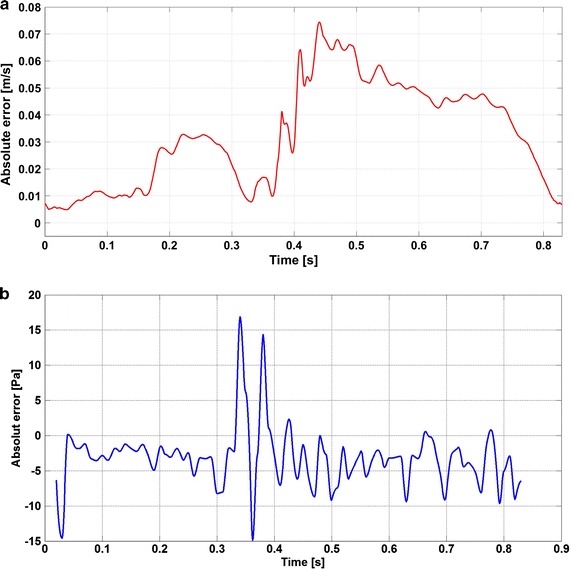
Velocity and pressure absolute error evaluation. The *plot* shows the absolute error between the simulations with and without wire for the velocity **a** and pressure **b**. The error is significant for the velocity **a** and negligible for the pressure **b** in comparison to the average blood pressure

The CFD study presented here allowed the estimation of the influence of the wire on intravascular measurements, as well as the impact of changing the wire tip orientation with respect to the vessel axis. Those comparisons are presented in Figs. 6, 7, 8, 9 and 10.

Figures 6 and 7 display 2D velocity fields of the configurations without wire (left column) and with wire (right column) on planes distant respectively 0, 5 and 10 mm from the guide wire tip. The velocity fields are presented for time t = 0.02 s (Fig. 2 marked as t_min_ corresponding to the minimum of the measured velocity in Fig. 6) and for time t = 0.44 s (Fig. 2 marked as t_max_) corresponding to the maximum of the measured velocity (see Fig. 7) of the pulsatile cycle having a period T = 0.83 s.

According to Fig. 6, the placement of the Doppler guide wire influences both the maximum velocity intensity and its position in the cutting plane. The disturbances are significant at the tip of the wire (0 mm distance) and decrease while moving away from it. However, while the maximum velocity tends to be similar in both cases, the maximum position still differs on the plane at 10 mm. Similar results were observed for the highest measured velocity (Fig. 7).

In order to perform proper maximum velocity measurements, the recommendation for the physicians is to insert the probe in the middle of the vessel and parallel to its axis. Therefore, the influence of a misalignment of the probe on the measured value was estimated with four additional simulations. The computations were based on the same inlet velocity condition for all cases. For the configuration “with wire”, 5 CFD simulations were performed; one in the reference orientation (named also as 0°) and four wire angles −10°, −5°, +5° and +10° compared to the reference orientation. The reference and ±10° orientations can be seen in the Fig. 8 and the final outcomes are presented in Fig. 9. It can be noticed that the wire orientation has a noticeable influence on the measured velocity values. The four orientations (−10°, −5°, +5° and +10°) present similar values (the four lines almost overlap), which are however significantly different from the reference at 0°. This can be explained by the geometry configuration: as presented in Fig. 8, the region where the maximum velocity was computed does not always cover the centre of the vessel (Fig. 8b) as well as the position of the volume slice coordinates (Fig. 8a).

In order to visualize the error that is introduced by the wire inside the vessel, a comparison between the configurations with and without wire is presented in Fig. 10a: for the velocity, it shows that the absolute error is almost ¼ of the maximum peak velocity and cannot be considered negligible.

The pressure absolute error caused by ComboWire^®^ is depicted in Fig. 10b: as it can be seen it is negligible compared to the average blood pressure values (10–16 kPa).

## Discussion

**Fig. 11:**
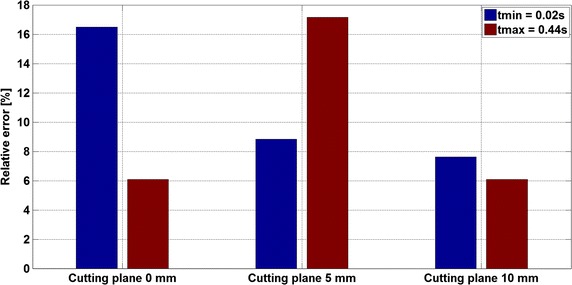
Result analysis—peak velocity relative errors. The figure presents the relative error of the peak velocity between the case with and without wire and cutting planes 0, 5 and 10 mm far from the guide wire tip. They are presented for the time step t_min_ = 0.02 s (*blue bars*) and t_max_ = 0.44 s (*red bars*)

This numerical study aims at assessing the influence of a catheter on the measure itself on the realistic 3D vessels geometry. The 3D CFD simulations were performed for the configuration with and without wire. Velocity fields for both configurations have been compared on cutting planes at distances ranging from 0 to 10 mm from the probe tip. Numerical simulations show that, even in this case where the ratio catheter diameter over vessel diameter is about 0.15, the influence of the probe on the velocity measurements is important, modifying the maximum velocity and inducing a shift of the peak position (Figs. 6, 7, 8, 9). As a result, placing the measured volume at 5.2 mm from the probe tip may not be sufficient to have a “clean measure”. As depicted in Fig. 11, there is still significant difference between the cases with and without wire. Depending on the flow rate, a significant relative error (e.g. relative difference between maximum velocities) for the simulated velocity peak varies from 17% at 5 mm cutting plane to 6% for 10 mm cutting plane with the highest flow rate.

**Fig. 12:**
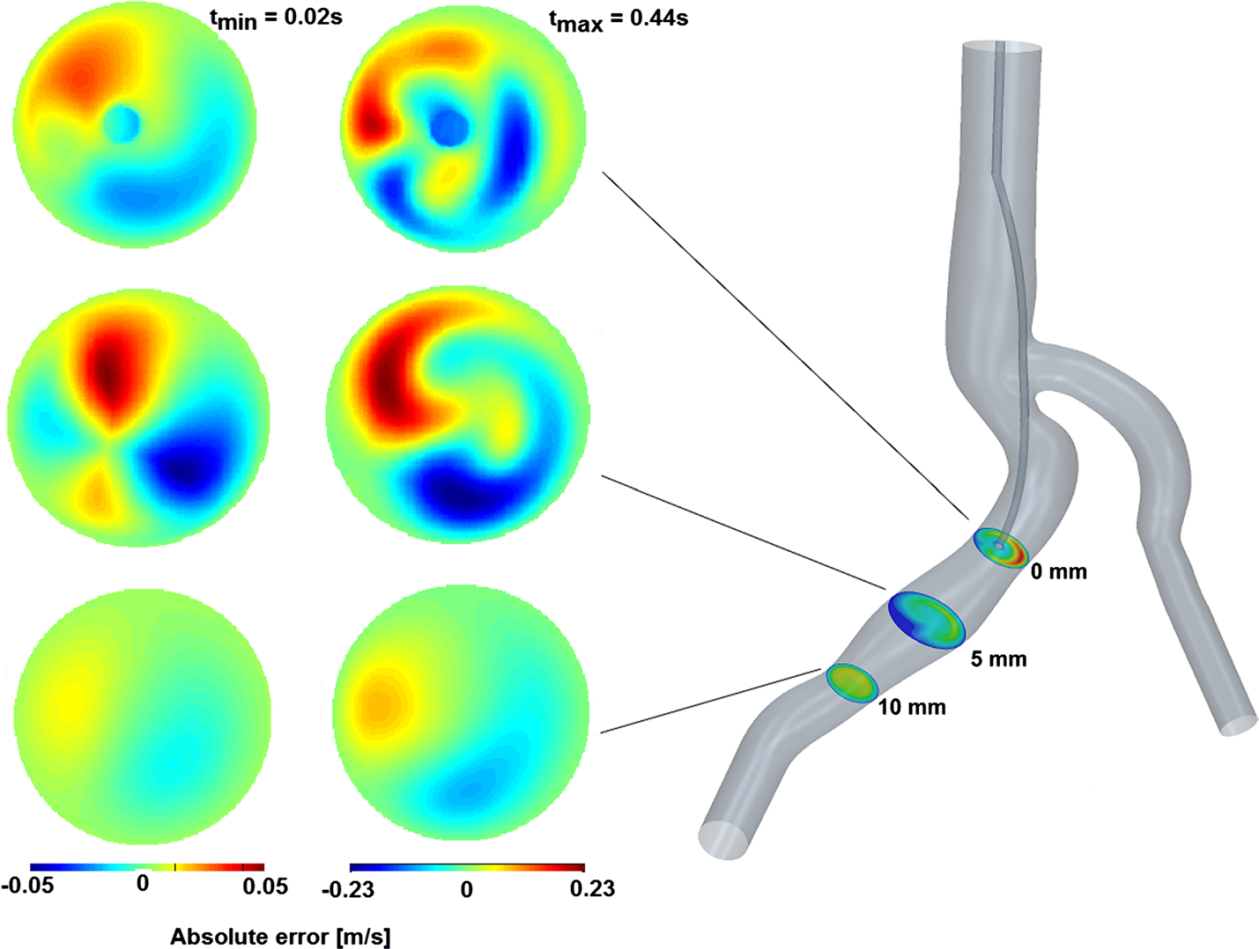
Result analysis—absolute errors on the sections. The absolute error of the velocity fields between the cases with and without wire is given. The error is calculated on three cutting planes at 0, 5 and 10 mm ahead of the guide wire tip. The results visualize the time step t_min_ = 0.02 s (*left column*) and t_max_ = 0.44 s (*right column*)

It can be concluded that the maximum velocity and the peak position are modified by the presence of the wire and even on a plane 10 mm far from the probe tip some discrepancies are still visible (see Fig. 12).

As human vasculature is complex, the diameter of vessel can vary from µm (capillaries) to cm (aorta) and wire interference is strongly influenced by the ratio “guide-wire diameter” over “vessel diameter” and should be taken into account to correct the data.

**Fig. 13:**
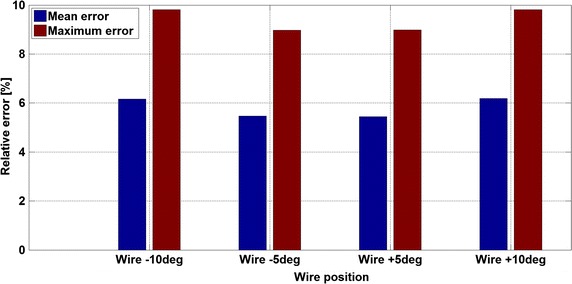
Result analysis—peak velocity relative errors for different probe tip angle

The additional simulations (Figs. 9, 13) run to investigate the effect of the misalignment of probe wire with the vessel axes (e.g. the cylindrical volume slice does not entirely cover the vessel) show that even a small misalignment angle may give a misleading measure (Fig. 9). The explanation can be found in measure methodology of the Doppler wire (Fig. 8): a different probe tip orientation changes the direction of the insonation beam (Fig. 8a) and the new volume slice covers a different vessel volume (Fig. 8b). The peak velocity errors (Fig. 13) are comprised between 5 and 10% and there is almost no difference between negative and positive orientations of the probe tip (e.g. ±5° and ±10° return the same peak velocity). Similar study with comparable conclusions was carried out in [19].

In order to have plausible inlet conditions, a Newton’s iterative algorithm has been developed to fit the measured velocity (e.g. the peak velocity in the vessel). It is shown that its convergence is fast and requires up to 4 iterations to fit the measurements.

It should be underlined that this study has been carried out for one particular geometry using only one measuring wire. It would be interesting for the future to study different geometries: bigger and smaller vessel diameters and more complex geometries. In addition, in order to access the desired vessel, physicians need to use a set of catheters before introducing the measuring device itself as those catheters are bigger than the probe wire. Their influence on the downstream flow could be analysed with CFD simulations.

In future, we expect to perform in vitro experiments with different type of vessel geometries, different type of guide wires in order to gather experimental data such as pressure and flow rate to validate CFD simulations.

## Conclusions

This study evidenced that the Doppler probe wire sampling volume at 5.2 mm from the probe tip interferes with the hemodynamic measurements. Simulations reveal that even at 10 mm far from the probe tip, its influence cannot be neglected. The error introduced by the probe varies between 6 and 17% for the peak velocity. Moreover, the misalignment error in a range of ±10° has also noticeable effect with a peak velocity error of about 6%. However, the pressure drop is not significantly altered and the pressure changes are below 15 Pa. Thus, physicians should take into account the disturbance phenomena induced by the presence of a catheter, mainly when the velocity is involved in the decision process and the ratio catheter diameter to vessel diameter is not small enough (Additional files 1, 2, 3, 4, 5, 6 and 7).

## Additional files

**Additional file 1.** Fourier coefficient decomposition for the first guess of the Newton’s method.

**Additional file 2.** java macro to be used with Star-CCM+ for inlet condition search (all Newton’s iterations except first).

**Additional file 3.** java macro to be used with Star-CCM+ for inlet condition search (first Newton’s iteration only).

**Additional file 4.** Matlab script to iteratively run the CFD simulations with updated inlet velocity for Newton’s method.

**Additional file 5.** HTML file summarizing the parameters used in Star-CCM to run the simulations.

**Additional file 6.** Geometry in parasolid file format, for configuration “without wire”.

**Additional file 7.** Geometry in parasolid file format, for configuration “with wire”.

## Abbreviations

QCA: quantitative coronary angiography
D: diameter (m)
R_1_,r: radius (m)
Re: Reynolds number (−)
Re_local_: local Reynolds number (−)
Re_max_: maximum Reynolds number (−)
ν: kinematic viscosity (m^2^/s)
υ_local_: local kinematic viscosity (m^2^/s)
u_max_: maximum value of inlet velocity (m/s)
ρ: density (kg/m^3^)
$$\dot{\gamma }$$: shear rate (s^−1^)
Hct: hematocrit level (%)
*μ*_0_: dynamic viscosity at zero shear rate (Pa s)
*μ*_∞_: dynamic viscosity at infinite shear rate (Pa s)
λ: relaxation time parameter (s)
*μ*: dynamic apparent viscosity (Pa s)
n: dimensionless coefficient (−)
*τ*: shear stress (Pa)
L_e_: entrance length (m)
p: pressure (Pa)
f: external volume force (m/s^2^)
t: time (s)
u(t): velocity in time (m/s)
e: error function (m/s)
e’: first derivative of the error function (−)
${\hat{\text{u}}}_{{{\text{sim}}.}} \left( {\text{t}} \right)$: is the simulated peak velocity in the volume slice (m/s)
${\hat{\text{u}}}_{{{\text{mes}}.}} \left( {\text{t}} \right)$: is the measured peak velocity in the volume slice (m/s)
