# Supplementary material for: Hemodynamic numerical simulations of the disturbance due to intracoronary flow measurements by a Doppler guide wire
Source: Biomed Eng Online. 2016 Oct 10;15:113. doi: 10.1186/s12938-016-0234-6 (PMC5490210; doi:10.1186/s12938-016-0234-6)
Supplement: Supplementary file 5 — Additional file 5. HTML file summarizing the parameters used in Star-CCM to run the simulations. [file 12938_2016_234_MOESM5_ESM.html]

Patient\_2\_real\_position\_wire


## Summary Report: Patient\_2\_real\_position\_wire

|  |
| --- |
| **Session Summary** |
| Date | 13-juil.-2016 15:06:06 |
| Simulation | G:\data\_article\starccm\_sim\Patient\_2\_real\_position\_wire.sim |
| File size | 45 MB |
| Number of Partitions | 4 |
| Number of Restored Partitions | 4 |
| **Software Summary** |
| Version | BuildArch: win64  BuildEnv: intel12.1  ReleaseDate: Fri Jan 24 18:36:08 UTC 2014  ReleaseNumber: 9.02.005 |
| MPI Version | Platform Computing MPI-8.3.0.3 |
| **Hardware Summary** |
| Hosts | Controller: PC-kamil2  Number of Workers: 3  Worker[1]: PC-kamil2  Worker[2]: PC-kamil2  Worker[3]: PC-kamil2 |

### Simulation Properties

|  |  |  |
| --- | --- | --- |
| 1 **Patient\_2\_real\_position\_wire** |  |  |
| +-1 Tags |  |  |
| +-2 Contacts |  |  |
| | `-1 Part Contact |  |  |
| |   `-1 In Place Contact | Tags | [] |
| | | Meta Data | {} |
| +-3 Filters |  |  |
| +-4 3D-CAD Models |  |  |
| | `-1 **3D-CAD Model 1** |  |  |
| +-5 Parts |  |  |
| | +-1 **Block** | Tags | [] |
| | | | | Meta Data | {} |
| | | | | Index | 14 |
| | | | | Region | [] |
| | | | | Contacts | [] |
| | | | | Descriptions | [Ljava.lang.String;@61cb3eab |
| | | | | Face count | 12 |
| | | | | Coordinate System | Laboratory |
| | | | | Corner 1 | [0.0073145064525306225, -0.0053525264374911785, -0.0016332351369783282] m,m,m |
| | | | | Corner 2 | [0.013603001832962036, 0.003576182760298252, 0.0014110029442235827] m,m,m |
| | | +-1 Surfaces |  |  |
| | | | `-1 **Block Surface** | Tags | [] |
| | | | | Meta Data | {} |
| | | | | Boundary | [] |
| | | `-2 Curves |  |  |
| | |   `-1 **Block Curve** | Tags | [] |
| | | | Feature Curve | [] |
| | +-2 **Cylinder** | Tags | [] |
| | | | | Meta Data | {} |
| | | | | Index | 9 |
| | | | | Region | [] |
| | | | | Contacts | [] |
| | | | | Descriptions | [Ljava.lang.String;@28f0b741 |
| | | | | Face count | 384 |
| | | | | Coordinate System | Laboratory |
| | | | | Start Coordinate | [0.0114341, -0.0105728, 0.0] m,m,m |
| | | | | End Coordinate | [0.0114341, -0.0095728, 0.0] m,m,m |
| | | | | Radius | 0.002 m |
| | | +-1 Surfaces |  |  |
| | | | `-1 **Cylinder Surface** | Tags | [] |
| | | | | Meta Data | {} |
| | | | | Boundary | [] |
| | | `-2 Curves |  |  |
| | |   `-1 **Cylinder Curve** | Tags | [] |
| | | | Feature Curve | [] |
| | +-3 **Cylinder+10** | Tags | [] |
| | | | | Meta Data | {} |
| | | | | Index | 17 |
| | | | | Region | [] |
| | | | | Contacts | [] |
| | | | | Descriptions | [Ljava.lang.String;@538f2166 |
| | | | | Face count | 384 |
| | | | | Coordinate System | Laboratory |
| | | | | Start Coordinate | [0.012351, -0.0099938, 0.0] m,m,m |
| | | | | End Coordinate | [0.012351, -0.0089938, 0.0] m,m,m |
| | | | | Radius | 0.002 m |
| | | +-1 Surfaces |  |  |
| | | | +-1 **Cylinder Surface** | Tags | [] |
| | | | | | Meta Data | {} |
| | | | | | Boundary | [] |
| | | | `-2 **Cylinder Surface 2** | Tags | [] |
| | | | | Meta Data | {} |
| | | | | Boundary | [] |
| | | `-2 Curves |  |  |
| | |   +-1 **Cylinder Curve** | Tags | [] |
| | |   | | Feature Curve | [] |
| | |   `-2 **Cylinder Curve 2** | Tags | [] |
| | | | Feature Curve | [] |
| | +-4 **Cylinder-10** | Tags | [] |
| | | | | Meta Data | {} |
| | | | | Index | 18 |
| | | | | Region | [] |
| | | | | Contacts | [] |
| | | | | Descriptions | [Ljava.lang.String;@68995e20 |
| | | | | Face count | 384 |
| | | | | Coordinate System | Laboratory |
| | | | | Start Coordinate | [0.0105172, -0.0099938, 0.0] m,m,m |
| | | | | End Coordinate | [0.0105172, -0.0089938, 0.0] m,m,m |
| | | | | Radius | 0.002 m |
| | | +-1 Surfaces |  |  |
| | | | +-1 **Cylinder Surface** | Tags | [] |
| | | | | | Meta Data | {} |
| | | | | | Boundary | [] |
| | | | `-2 **Cylinder Surface 2** | Tags | [] |
| | | | | Meta Data | {} |
| | | | | Boundary | [] |
| | | `-2 Curves |  |  |
| | |   +-1 **Cylinder Curve** | Tags | [] |
| | |   | | Feature Curve | [] |
| | |   `-2 **Cylinder Curve 2** | Tags | [] |
| | | | Feature Curve | [] |
| | +-5 **Intersect** | Tags | [] |
| | | | | Meta Data | {} |
| | | | | Index | 12 |
| | | | | Region | [Region] |
| | | | | Contacts | [Subtract] |
| | | | | Descriptions | [Ljava.lang.String;@2cf2bc86 |
| | | | | Face count | 2176 |
| | | +-1 Surfaces |  |  |
| | | | +-1 **interface\_cyl** | Tags | [] |
| | | | | | Meta Data | {} |
| | | | | | Boundary | [] |
| | | | +-2 **PartSurface\_Intersect\_Subtract** | Tags | [] |
| | | | | | Meta Data | {} |
| | | | | | Boundary | [Region: Intersect.PartSurface\_Intersect\_Subtract] |
| | | | `-3 **wall\_cyl** | Tags | [] |
| | | | | Meta Data | {} |
| | | | | Boundary | [Region: Intersect.wall\_cyl] |
| | | `-2 Curves |  |  |
| | |   +-1 **Cylinder Curve** | Tags | [] |
| | |   | | Feature Curve | [Region:Feature Curve] |
| | |   +-2 **Default** | Tags | [] |
| | |   | | Feature Curve | [Region:Feature Curve] |
| | |   `-3 **Intersection** | Tags | [] |
| | | | Feature Curve | [Region:Feature Curve] |
| | +-6 **Subtract** | Tags | [] |
| | | | | Meta Data | {} |
| | | | | Index | 13 |
| | | | | Region | [Region] |
| | | | | Contacts | [Intersect] |
| | | | | Descriptions | [Ljava.lang.String;@67d94765 |
| | | | | Face count | 287146 |
| | | +-1 Surfaces |  |  |
| | | | +-1 **inlet** | Tags | [] |
| | | | | | Meta Data | {} |
| | | | | | Boundary | [Region: Subtract.inlet] |
| | | | +-2 **interface\_vessel** | Tags | [] |
| | | | | | Meta Data | {} |
| | | | | | Boundary | [] |
| | | | +-3 **outletBig** | Tags | [] |
| | | | | | Meta Data | {} |
| | | | | | Boundary | [Region: Subtract.outletBig] |
| | | | +-4 **outletSmall** | Tags | [] |
| | | | | | Meta Data | {} |
| | | | | | Boundary | [Region: Subtract.outletSmall] |
| | | | +-5 **PartSurface\_Subtract\_Intersect** | Tags | [] |
| | | | | | Meta Data | {} |
| | | | | | Boundary | [Region: Subtract.PartSurface\_Subtract\_Intersect] |
| | | | `-6 **wall** | Tags | [] |
| | | | | Meta Data | {} |
| | | | | Boundary | [Region: Subtract.wall] |
| | | `-2 Curves |  |  |
| | |   +-1 **Cylinder Curve** | Tags | [] |
| | |   | | Feature Curve | [Region:Feature Curve] |
| | |   +-2 **Edges** | Tags | [] |
| | |   | | Feature Curve | [Region:Feature Curve] |
| | |   `-3 **Intersection** | Tags | [] |
| | | | Feature Curve | [Region:Feature Curve] |
| | `-7 **Vessel1\_wire\_new** | Tags | [] |
| |   | | Meta Data | {} |
| |   | | Index | 8 |
| |   | | Region | [] |
| |   | | Contacts | [] |
| |   | | Descriptions | [Ljava.lang.String;@6cc9f4f1 |
| |   | | Face count | 287602 |
| |   +-1 Surfaces |  |  |
| |   | `-1 **Faces** | Tags | [] |
| |   | | Meta Data | {} |
| |   | | Boundary | [] |
| |   `-2 Curves |  |  |
| |     `-1 **Edges** | Tags | [] |
| | | Feature Curve | [] |
| +-6 Operations |  |  |
| +-7 Descriptions | Number of children | 1 |
| | `-1 Root | Described Parts | Vessel1\_wire\_new, Cylinder, Intersect, Subtract, Block, Cylinder+10, Cylinder-10 |
| +-8 Continua | Continua | 2 |
| | +-1 **Mesh 1** | Regions | [Region] |
| | | | | Interfaces | [Intersect/Subtract] |
| | | | | OOC translation | false |
| | | | | Verbose Output | false |
| | | | | Per-Region Meshing | false |
| | | | | Enable Parallel Meshing | false |
| | | | | Interpolation Option | Nearest neighbor |
| | | +-1 Models |  |  |
| | | | +-1 Prism Layer Mesher | Stretching Function | Geometric Progression |
| | | | | | Stretching Mode | Stretch Factor |
| | | | | | Gap Fill Percentage | 25.0 |
| | | | | | Minimum Thickness Percentage | 10.0 |
| | | | | | Layer Reduction Percentage | 50.0 |
| | | | | | Boundary March Angle | 50.0 |
| | | | | | Concave Angle Limit | 0.0 |
| | | | | | Convex Angle Limit | 360.0 |
| | | | | | Near Core Layer Aspect Ratio | 0.0 |
| | | | | | Generate Standard Cells Only | false |
| | | | | | Improve Subsurface Quality | true |
| | | | +-2 Surface Remesher | Do curvature refinement | true |
| | | | | | Do proximity refinement | true |
| | | | | | Do compatibility refinement | false |
| | | | | | Retain geometric features | true |
| | | | | | Create aligned meshes | true |
| | | | | | Minimum Face Quality | 0.05 |
| | | | | | Enable automatic surface repair | true |
| | | | `-3 Trimmer | Coordinate System | Laboratory |
| | | | | Do mesh alignment | false |
| | | | | Template mesh type | Hexahedra |
| | | | | Template mesh growth type | Simple |
| | | | | Run Optimizer | true |
| | | +-2 Reference Values |  |  |
| | | | +-1 Base Size | Value | 1.0E-4 m |
| | | | +-2 Automatic Surface Repair | Connected surface count limit | None |
| | | | | | | Connected surface size limits | None |
| | | | | +-1 Minimum Proximity | Minimum Proximity | 0.05 |
| | | | | `-2 Minimum Quality | Minimum Quality | 0.01 |
| | | | +-3 CAD Projection | Project to CAD | true |
| | | | +-4 Maximum Cell Size | Size type | Relative to base |
| | | | | `-1 Relative Size | Percentage of Base | 75.0 |
| | | | | | Absolute Size | 7.500000000000001E-5 m |
| | | | +-5 Maximum Core/Prism Transition Ratio | Limit cell size by prism layer thickness | false |
| | | | +-6 Number of Prism Layers | Number of Prism Layers | 3 |
| | | | +-7 Prism Layer Stretching | Prism Layer Stretching | 1.3 |
| | | | +-8 Prism Layer Thickness | Size type | Relative to base |
| | | | | `-1 Relative Size | Percentage of Base | 20.0 |
| | | | | | Absolute Size | 2.0E-5 m |
| | | | +-9 Surface Curvature | Enable curvature deviation distance | false |
| | | | | `-1 Basic Curvature | # Pts/circle | 36.0 |
| | | | +-10 Surface Growth Rate | Surface Growth Rate | 1.3 |
| | | | +-11 Surface Proximity | Search Floor | 0.0 m |
| | | | | | # Points in gap | 2.0 |
| | | | | | Enable Search Ceiling | false |
| | | | +-12 Surface Size | Relative/Absolute | Relative to base |
| | | | | | | Size Method | Min and Target |
| | | | | +-1 Relative Minimum Size | Percentage of Base | 50.0 |
| | | | | | | Absolute Size | 5.0E-5 m |
| | | | | `-2 Relative Target Size | Percentage of Base | 100.0 |
| | | | | | Absolute Size | 1.0E-4 m |
| | | | `-13 Template Growth Rate | Default Growth Rate | Medium |
| | | | | Boundary Growth Rate | Fast |
| | | `-3 Volumetric Controls |  |  |
| | `-2 **Physics 1** | Regions | [Region] |
| |   | | Interfaces | [Intersect/Subtract] |
| |   +-1 Models |  |  |
| |   | +-1 Constant Density |  |  |
| |   | +-2 Gradients | Gradient Method | Hybrid Gauss-LSQ |
| |   | | | Limiter Method | Venkatakrishnan |
| |   | | | Custom Accuracy Level Selector | 2.0 |
| |   | | | Verbose | false |
| |   | | | Least-Squares Quality Criterion | true |
| |   | | | Flat Cells Curvature Criterion | true |
| |   | | | Cell Skewness Criterion | true |
| |   | | | Chevron-Cell Criterion | true |
| |   | | | Least-Squares Tensor Minimum Eigenvalues Ratio | 0.1 |
| |   | | | Normalized Flat Cells Curvature Factor | 1.0 |
| |   | | | Maximum safe (positive) skewness angle (deg.) | 75.0 |
| |   | | | Minimum unsafe (positive) skewness angle (deg.) | 88.0 |
| |   | | | Use TVB Gradient Limiting | false |
| |   | | | Acceptable Field Variation (Factor) | 0.05 |
| |   | +-3 Implicit Unsteady |  |  |
| |   | +-4 Laminar |  |  |
| |   | +-5 Liquid |  |  |
| |   | | `-1 **Blood** | Database Material | Blood (Blood) [Standard/Liquids] |
| |   | |   `-1 Material Properties |  |  |
| |   | |     +-1 Density | Method | Constant |
| |   | |     | `-1 Constant | Value | 1055.6 kg/m^3 |
| |   | |     `-2 Dynamic Viscosity | Method | Non-Newtonian Generalized Carreau-Yasuda Fluid |
| |   | |       `-1 Non-Newtonian Generalized Carreau-Yasuda Fluid | Power Constant | 0.39729 |
| |   | | | a Parameter | 2.0 |
| |   | | | Zero Shear Viscosity | 0.021732 Pa-s |
| |   | | | Infinite Shear Viscosity | 0.0026094 Pa-s |
| |   | | | Relaxation Time | 1.4756 s |
| |   | | | Viscosity Under-Relaxation Factor | 0.7 |
| |   | +-6 Segregated Flow | Minimum Absolute Pressure | 1000.0 Pa |
| |   | | | Flow Boundary Diffusion | true |
| |   | | | Unsteady Flux Dissipation Corrections | false |
| |   | | | Limiting Acoustic-CFL | 1.0 |
| |   | | | Secondary Gradients | On |
| |   | | | Convection | 2nd-order |
| |   | | | Delta-V Dissipation | Off |
| |   | `-7 Three Dimensional |  |  |
| |   +-2 Reference Values |  |  |
| |   | `-1 Reference Pressure | Value | 101325.0 Pa |
| |   `-3 Initial Conditions |  |  |
| |     +-1 Pressure | Method | Constant |
| |     | `-1 Constant | Value | 101325.0 Pa |
| |     `-2 Velocity | Method | Constant |
| |       | | Coordinate System | Laboratory |
| |       `-1 Constant | Value | [0.0, 0.0, 0.0] m/s |
| +-9 Regions | Regions | 1 |
| | `-1 **Region** | Index | 1 |
| |   | | Mesh Continuum | Mesh 1 |
| |   | | Physics Continuum | Physics 1 |
| |   | | Parts | [Intersect, Subtract] |
| |   | | Type | Fluid Region |
| |   +-1 Boundaries | Boundaries | 9 |
| |   | +-1 **Intersect.PartSurface\_Intersect\_Subtract** | Index | 4 |
| |   | | | | Interfaces | Intersect/Subtract |
| |   | | | | Part Surfaces | [Intersect.PartSurface\_Intersect\_Subtract] |
| |   | | | | Type | Wall |
| |   | | +-1 Mesh Conditions |  |  |
| |   | | | +-1 Custom Boundary Growth Rate | Custom Boundary Growth Rate | Disabled |
| |   | | | +-2 Custom Surface Curvature | Custom curvature | Use Continuum Values |
| |   | | | +-3 Custom Surface Proximity | Custom proximity | Use Continuum Values |
| |   | | | +-4 Custom Surface Size | Custom surface size | Disabled |
| |   | | | +-5 Customize Prism Mesh | Customize Prism Mesh | Use Default Values |
| |   | | | `-6 Customize Surface Remeshing | Disable surface remeshing | Disabled |
| |   | | `-2 Physics Conditions |  |  |
| |   | |   +-1 Shear Stress Specification | Method | No-Slip |
| |   | |   `-2 Tangential Velocity Specification | Method | None |
| |   | | | Reference Frame | Relative To Mesh |
| |   | +-2 **Intersect.PartSurface\_Intersect\_Subtract [Intersect/Subtract]** | Index | 11 |
| |   | | | Interfaces |  |
| |   | | | Part Surfaces | [] |
| |   | | | Type | Internal Interface Boundary |
| |   | | | Parent Interface | Intersect/Subtract |
| |   | +-3 **Intersect.wall\_cyl** | Index | 2 |
| |   | | | | Interfaces |  |
| |   | | | | Part Surfaces | [Intersect.wall\_cyl] |
| |   | | | | Type | Wall |
| |   | | +-1 Mesh Conditions |  |  |
| |   | | | +-1 Custom Boundary Growth Rate | Custom Boundary Growth Rate | Disabled |
| |   | | | +-2 Custom Surface Curvature | Custom curvature | Use Continuum Values |
| |   | | | +-3 Custom Surface Proximity | Custom proximity | Use Continuum Values |
| |   | | | +-4 Custom Surface Size | Custom surface size | Disabled |
| |   | | | +-5 Customize Prism Mesh | Customize Prism Mesh | Use Default Values |
| |   | | | `-6 Customize Surface Remeshing | Disable surface remeshing | Disabled |
| |   | | `-2 Physics Conditions |  |  |
| |   | |   +-1 Shear Stress Specification | Method | No-Slip |
| |   | |   `-2 Tangential Velocity Specification | Method | None |
| |   | | | Reference Frame | Relative To Mesh |
| |   | +-4 **Subtract.inlet** | Index | 9 |
| |   | | | | Interfaces |  |
| |   | | | | Part Surfaces | [Subtract.inlet] |
| |   | | | | Type | Velocity Inlet |
| |   | | +-1 Mesh Conditions |  |  |
| |   | | | +-1 Custom Boundary Growth Rate | Custom Boundary Growth Rate | Disabled |
| |   | | | +-2 Custom Surface Curvature | Custom curvature | Use Continuum Values |
| |   | | | +-3 Custom Surface Proximity | Custom proximity | Use Continuum Values |
| |   | | | +-4 Custom Surface Size | Custom surface size | Disabled |
| |   | | | +-5 Customize Prism Mesh | Customize Prism Mesh | Use Default Values |
| |   | | | `-6 Customize Surface Remeshing | Disable surface remeshing | Disabled |
| |   | | +-2 Physics Conditions |  |  |
| |   | | | +-1 Flow Direction Specification | Method | Boundary-Normal |
| |   | | | +-2 Reference Frame Specification | Option | Lab Frame |
| |   | | | `-3 Velocity Specification | Method | Magnitude + Direction |
| |   | | `-3 Physics Values |  |  |
| |   | |   `-1 Velocity Magnitude | Method | Table (time) |
| |   | |     `-1 Table (time) | Table | velm |
| |   | | | Table: Data | velocity |
| |   | | | Table: Time | time |
| |   | | | Interpolation | Spline |
| |   | +-5 **Subtract.outletBig** | Index | 8 |
| |   | | | | Interfaces |  |
| |   | | | | Part Surfaces | [Subtract.outletBig] |
| |   | | | | Type | Pressure Outlet |
| |   | | +-1 Mesh Conditions |  |  |
| |   | | | +-1 Custom Boundary Growth Rate | Custom Boundary Growth Rate | Disabled |
| |   | | | +-2 Custom Surface Curvature | Custom curvature | Use Continuum Values |
| |   | | | +-3 Custom Surface Proximity | Custom proximity | Use Continuum Values |
| |   | | | +-4 Custom Surface Size | Custom surface size | Disabled |
| |   | | | +-5 Customize Prism Mesh | Customize Prism Mesh | Use Default Values |
| |   | | | `-6 Customize Surface Remeshing | Disable surface remeshing | Disabled |
| |   | | +-2 Physics Conditions |  |  |
| |   | | | +-1 Backflow Direction Specification | Method | Boundary-Normal |
| |   | | | +-2 Pressure Jump Option | Option | None |
| |   | | | +-3 Pressure Specification | Pressure specification | Environmental |
| |   | | | +-4 Reference Frame Specification | Option | Lab Frame |
| |   | | | `-5 Target Mass Flow Option | Target Mass Flow Option | Disabled |
| |   | | `-3 Physics Values |  |  |
| |   | |   `-1 Pressure | Method | Constant |
| |   | |     `-1 Constant | Value | 101325.0 Pa |
| |   | +-6 **Subtract.outletSmall** | Index | 7 |
| |   | | | | Interfaces |  |
| |   | | | | Part Surfaces | [Subtract.outletSmall] |
| |   | | | | Type | Pressure Outlet |
| |   | | +-1 Mesh Conditions |  |  |
| |   | | | +-1 Custom Boundary Growth Rate | Custom Boundary Growth Rate | Disabled |
| |   | | | +-2 Custom Surface Curvature | Custom curvature | Use Continuum Values |
| |   | | | +-3 Custom Surface Proximity | Custom proximity | Use Continuum Values |
| |   | | | +-4 Custom Surface Size | Custom surface size | Disabled |
| |   | | | +-5 Customize Prism Mesh | Customize Prism Mesh | Use Default Values |
| |   | | | `-6 Customize Surface Remeshing | Disable surface remeshing | Disabled |
| |   | | +-2 Physics Conditions |  |  |
| |   | | | +-1 Backflow Direction Specification | Method | Boundary-Normal |
| |   | | | +-2 Pressure Jump Option | Option | None |
| |   | | | +-3 Pressure Specification | Pressure specification | Environmental |
| |   | | | +-4 Reference Frame Specification | Option | Lab Frame |
| |   | | | `-5 Target Mass Flow Option | Target Mass Flow Option | Disabled |
| |   | | `-3 Physics Values |  |  |
| |   | |   `-1 Pressure | Method | Constant |
| |   | |     `-1 Constant | Value | 101325.0 Pa |
| |   | +-7 **Subtract.PartSurface\_Subtract\_Intersect** | Index | 10 |
| |   | | | | Interfaces | Intersect/Subtract |
| |   | | | | Part Surfaces | [Subtract.PartSurface\_Subtract\_Intersect] |
| |   | | | | Type | Wall |
| |   | | +-1 Mesh Conditions |  |  |
| |   | | | +-1 Custom Boundary Growth Rate | Custom Boundary Growth Rate | Disabled |
| |   | | | +-2 Custom Surface Curvature | Custom curvature | Use Continuum Values |
| |   | | | +-3 Custom Surface Proximity | Custom proximity | Use Continuum Values |
| |   | | | +-4 Custom Surface Size | Custom surface size | Disabled |
| |   | | | +-5 Customize Prism Mesh | Customize Prism Mesh | Use Default Values |
| |   | | | `-6 Customize Surface Remeshing | Disable surface remeshing | Disabled |
| |   | | `-2 Physics Conditions |  |  |
| |   | |   +-1 Shear Stress Specification | Method | No-Slip |
| |   | |   `-2 Tangential Velocity Specification | Method | None |
| |   | | | Reference Frame | Relative To Mesh |
| |   | +-8 **Subtract.PartSurface\_Subtract\_Intersect [Intersect/Subtract]** | Index | 12 |
| |   | | | Interfaces |  |
| |   | | | Part Surfaces | [] |
| |   | | | Type | Internal Interface Boundary |
| |   | | | Parent Interface | Intersect/Subtract |
| |   | `-9 **Subtract.wall** | Index | 5 |
| |   |   | | Interfaces |  |
| |   |   | | Part Surfaces | [Subtract.wall] |
| |   |   | | Type | Wall |
| |   |   +-1 Mesh Conditions |  |  |
| |   |   | +-1 Custom Boundary Growth Rate | Custom Boundary Growth Rate | Disabled |
| |   |   | +-2 Custom Surface Curvature | Custom curvature | Use Continuum Values |
| |   |   | +-3 Custom Surface Proximity | Custom proximity | Use Continuum Values |
| |   |   | +-4 Custom Surface Size | Custom surface size | Disabled |
| |   |   | +-5 Customize Prism Mesh | Customize Prism Mesh | Use Default Values |
| |   |   | `-6 Customize Surface Remeshing | Disable surface remeshing | Disabled |
| |   |   `-2 Physics Conditions |  |  |
| |   |     +-1 Shear Stress Specification | Method | No-Slip |
| |   |     `-2 Tangential Velocity Specification | Method | None |
| |   | | Reference Frame | Relative To Mesh |
| |   +-2 Feature Curves | Feature Curves | 1 |
| |   | `-1 **Feature Curve** | Part Curves | [Intersect.Cylinder Curve, Intersect.Intersection, Intersect.Default, Subtract.Edges, Subtract.Cylinder Curve, Subtract.Intersection] |
| |   |   `-1 Mesh Conditions |  |  |
| |   |     `-1 Custom Surface Size | Custom surface size | Disabled |
| |   +-3 Mesh Conditions |  |  |
| |   | `-1 Customize Prism Mesh | Customize Prism Mesh | Use Default Values |
| |   +-4 Mesh Values |  |  |
| |   | `-1 Trimmer Wake Refinement |  |  |
| |   +-5 Physics Conditions |  |  |
| |   | +-1 Initial Condition Option | Option | Use Continuum Values |
| |   | +-2 Mass Source Option | Mass Source Option | Disabled |
| |   | `-3 Momentum Source Option | Momentum Source Option | None |
| |   `-6 Physics Values |  |  |
| |     +-1 Axis | Coordinate System | Laboratory |
| |     | | Origin | [0.0, 0.0, 0.0] m |
| |     | | Direction | [0.0, 0.0, 1.0] |
| |     `-2 Motion Specification | Motion | Stationary |
| | | Reference Frame | Lab Reference Frame |
| +-10 Interfaces | Verbosity | false |
| | | | Interfaces | 1 |
| | `-1 **Intersect/Subtract** | Enabled | true |
| |   | | Boundary-0 | Region: Intersect.PartSurface\_Intersect\_Subtract |
| |   | | Boundary-1 | Region: Subtract.PartSurface\_Subtract\_Intersect |
| |   | | Type | Internal Interface |
| |   | | Topology | In-place |
| |   | | Connectivity | Imprinted |
| |   | | Intersector | Default |
| |   +-1 Mesh Conditions |  |  |
| |   | +-1 Custom Boundary Growth Rate | Custom Boundary Growth Rate | Disabled |
| |   | +-2 Custom Surface Curvature | Custom curvature | Use Continuum Values |
| |   | +-3 Custom Surface Proximity | Custom proximity | Use Continuum Values |
| |   | +-4 Custom Surface Size | Custom surface size | Disabled |
| |   | +-5 Customize Surface Remeshing | Disable surface remeshing | Disabled |
| |   | `-6 Interface Prism Layer Option | Grow Prisms from Interface | false |
| |   `-2 Physics Values |  |  |
| |     `-1 Intersection | Tolerance | 0.05 |
| +-11 Derived Parts | Derived Parts | 7 |
| | +-1 **articlePlane** | Origin | [0.0115825638547, -0.00481484638547, 7.473281457334117E-5] m,m,m |
| | | | | Coordinate System | Laboratory |
| | | | | Normal | [0.0, 1.0, 0.0] m,m,m |
| | | | | Parts | [Region] |
| | | | | Section Mode | Single Section |
| | | | | Displayed Index | -1 |
| | | `-1 Single section | Offset | 0.0 m |
| | +-2 **articlePlane2** | Origin | [0.0115825638547, -0.00481484638547, 7.473281457334117E-5] m,m,m |
| | | | | Coordinate System | Laboratory |
| | | | | Normal | [0.0, 1.0, 0.0] m,m,m |
| | | | | Parts | [Region] |
| | | | | Section Mode | Single Section |
| | | | | Displayed Index | -1 |
| | | `-1 Single section | Offset | -0.005 m |
| | +-3 **articlePlane3** | Origin | [0.0115825638547, -0.00481484638547, 7.473281457334117E-5] m,m,m |
| | | | | Coordinate System | Laboratory |
| | | | | Normal | [0.0, 1.0, 0.0] m,m,m |
| | | | | Parts | [Region] |
| | | | | Section Mode | Single Section |
| | | | | Displayed Index | -1 |
| | | `-1 Single section | Offset | -0.01 m |
| | +-4 **measurePlane0** | Origin | [0.0115825638547, -0.00481484638547, 7.473281457334117E-5] m,m,m |
| | | | | Coordinate System | Laboratory |
| | | | | Normal | [0.0, 1.0, 0.0] m,m,m |
| | | | | Parts | [Region] |
| | | | | Section Mode | Single Section |
| | | | | Displayed Index | -1 |
| | | `-1 Single section | Offset | -0.0052 m |
| | +-5 **measurePlane10** | Origin | [0.0115825638547, -0.00481484638547, 7.473281457334117E-5] m,m,m |
| | | | | Coordinate System | Laboratory |
| | | | | Normal | [0.0, 1.0, 0.0] m,m,m |
| | | | | Parts | [Region] |
| | | | | Section Mode | Single Section |
| | | | | Displayed Index | -1 |
| | | `-1 Single section | Offset | -0.0047 m |
| | +-6 **plane section** | Origin | [0.0, 0.0, 0.0] m,m,m |
| | | | | Coordinate System | Laboratory |
| | | | | Normal | [0.0, 0.0, 1.0] m,m,m |
| | | | | Parts | [Region] |
| | | | | Section Mode | Single Section |
| | | | | Displayed Index | -1 |
| | | `-1 Single section | Offset | 0.0 m |
| | `-7 **point** | Parts | [Region] |
| | | Point | [0.0, 0.0, 0.0] m,m,m |
| | | Coordinate System | Laboratory->Cartesian 1 |
| | | Follow Motion | false |
| +-12 Solvers |  |  |
| | +-1 Implicit Unsteady | Solver Frozen | false |
| | | | Time-Step | 0.001 s |
| | | | Freeze Time | false |
| | | | Temporal Discretization | 2nd-order |
| | +-2 Partitioning | Solver Frozen | false |
| | | | Partitioning method | Per-Continuum |
| | `-3 Segregated Flow | Solver Frozen | false |
| |   | | Reconstruction Frozen | false |
| |   | | Reconstruction Zeroed | false |
| |   | | Temporary Storage Retained | false |
| |   | | Continuity Initialization | false |
| |   | | Continuity Initialization Iterations | 3 |
| |   | | Continuity Initialization Tolerance | 1.0E-6 |
| |   +-1 Velocity | Under-Relaxation Factor | 0.9 |
| |   | | | Dynamic Local Under-Relaxation | false |
| |   | +-1 Under-Relaxation Factor Ramp | Ramp Method | No Ramp |
| |   | `-2 AMG Linear Solver | Max Cycles | 30 |
| |   |   | | Verbosity | None |
| |   |   | | Parallel Migration Limit | 25 |
| |   |   | | Subdomain coarsening enabled | true |
| |   |   | | Enable direct-solver | false |
| |   |   | | Maximum direct-solver equations | 32 |
| |   |   | | Convergence Tolerance | 0.1 |
| |   |   | | Epsilon | 0.0 |
| |   |   | | Cycle Type | Flex Cycle |
| |   |   | | Group Size Control | Auto |
| |   |   | | Group Size | 4 |
| |   |   | | Relaxation Scheme | Gauss-Seidel |
| |   |   | | Acceleration method | None |
| |   |   | | Scaling | Disabled |
| |   |   `-1 Flex Cycle | Restriction Tolerance | 0.9 |
| |   | | Prolongation Tolerance | 0.5 |
| |   | | Sweeps | 1 |
| |   `-2 Pressure | Under-Relaxation Factor | 0.2 |
| |     | | Pressure Reference Location | Automatic Selection |
| |     +-1 Under-Relaxation Factor Ramp | Ramp Method | No Ramp |
| |     `-2 AMG Linear Solver | Max Cycles | 30 |
| |       | | Verbosity | None |
| |       | | Parallel Migration Limit | 25 |
| |       | | Subdomain coarsening enabled | true |
| |       | | Enable direct-solver | false |
| |       | | Maximum direct-solver equations | 32 |
| |       | | Convergence Tolerance | 0.1 |
| |       | | Epsilon | 0.0 |
| |       | | Cycle Type | V Cycle |
| |       | | Group Size Control | Auto |
| |       | | Group Size | 4 |
| |       | | Relaxation Scheme | Gauss-Seidel |
| |       | | Acceleration method | None |
| |       | | Scaling | Auto |
| |       `-1 V Cycle | Pre-Sweeps | 1 |
| | | Post-Sweeps | 1 |
| | | Max Levels | 50 |
| +-13 Stopping Criteria |  |  |
| | +-1 **Maximum Inner Iterations** | Enabled | true |
| | | | Maximum Inner Iterations | 25 |
| | | | Logical Rule | Or |
| | | | Criterion Satisfied | false |
| | +-2 **Maximum Physical Time** | Enabled | true |
| | | | Maximum Physical Time | 2.49 s |
| | | | Logical Rule | Or |
| | | | Criterion Satisfied | false |
| | +-3 **Maximum Steps** | Enabled | false |
| | | | Maximum Steps | 1000 |
| | | | Logical Rule | Or |
| | | | Criterion Satisfied | false |
| | `-4 **Stop File** | Enabled | true |
| | | Stop Inner Iterations | true |
| | | Path | ABORT |
| | | Logical Rule | Or |
| | | Criterion Satisfied | false |
| +-14 Solution Histories |  |  |
| +-15 Solution Views |  |  |
| | `-1 **Current Solution** | Iteration | 0 |
| | | Time Step | 0 |
| | | Solution Time | 0.0 |
| +-16 Reports | Reports | 6 |
| | +-1 **Average\_Local\_Re** | Units |  |
| | | | Scalar Field Function | Local\_Reynolds |
| | | | Parts | [Region] |
| | | | Smooth Values | false |
| | +-2 **Max\_Local\_Re** | Units |  |
| | | | Scalar Field Function | Local\_Reynolds |
| | | | Parts | [Region, Region: Intersect.PartSurface\_Intersect\_Subtract, Region: Intersect.PartSurface\_Intersect\_Subtract [Intersect/Subtract], Region: Intersect.wall\_cyl, Region: Subtract.inlet, Region: Subtract.outletBig, Region: Subtract.outletSmall, Region: Subtract.PartSurface\_Subtract\_Intersect, Region: Subtract.PartSurface\_Subtract\_Intersect [Intersect/Subtract], Region: Subtract.wall] |
| | | | Smooth Values | false |
| | +-3 **Max\_Vel\_iteration** | Units | m/s |
| | | | Scalar Field Function | Velocity: Magnitude |
| | | | Parts | [Intersect, Intersect.interface\_cyl, Intersect.PartSurface\_Intersect\_Subtract, Intersect.wall\_cyl] |
| | | | Smooth Values | false |
| | +-4 **Maximum 1** | Units | m/s |
| | | | Scalar Field Function | Velocity: Magnitude |
| | | | Parts | [Intersect, Intersect.interface\_cyl, Intersect.PartSurface\_Intersect\_Subtract, Intersect.wall\_cyl] |
| | | | Smooth Values | false |
| | +-5 **MaxPoint** | Units | Pa |
| | | | Scalar Field Function | Absolute Pressure |
| | | | Parts | [point] |
| | | | Smooth Values | false |
| | `-6 **maxPressureReport** | Units | Pa |
| | | Scalar Field Function | Absolute Pressure |
| | | Parts | [] |
| | | Smooth Values | false |
| +-17 Monitors | Monitors | 14 |
| | | | Monitors To Print | [Continuity, X-momentum, Y-momentum, Z-momentum, Maximum 1 Monitor, Average\_Local\_Re Monitor, Max\_Local\_Re Monitor, Max\_Vel\_iteration Monitor, Average\_Local\_Re Monitor 2, Max\_Local\_Re Monitor 2] |
| | | | Output Direction | Horizontal |
| | | | Heading Print Frequency | 10 |
| | +-1 **Average\_Local\_Re Monitor** | Report | Average\_Local\_Re |
| | | | | Enabled | true |
| | | | | Trigger | Iteration |
| | | | | Normalization Option | Off |
| | | | | Maximum Plot Samples | 5000 |
| | | `-1 Iteration Frequency | Iteration Frequency | 1 |
| | | | Start Iteration | 0 |
| | | | Enable Stop | false |
| | | | Stop Iteration | 0 |
| | +-2 **Average\_Local\_Re Monitor 2** | Report | Average\_Local\_Re |
| | | | | Enabled | true |
| | | | | Trigger | Time Step |
| | | | | Normalization Option | Off |
| | | | | Maximum Plot Samples | 5000 |
| | | `-1 Time-Step Frequency | Time-step Frequency | 1 |
| | | | Start Time-step | 0 |
| | | | Enable Stop | false |
| | | | Stop Time-step | 0 |
| | +-3 **Field Sum 1** | Parts | [Region, Region: Intersect.PartSurface\_Intersect\_Subtract, Region: Intersect.PartSurface\_Intersect\_Subtract [Intersect/Subtract], Region: Intersect.wall\_cyl, Region: Subtract.inlet, Region: Subtract.outletBig, Region: Subtract.outletSmall, Region: Subtract.PartSurface\_Subtract\_Intersect, Region: Subtract.PartSurface\_Subtract\_Intersect [Intersect/Subtract], Region: Subtract.wall] |
| | | | | Enabled | true |
| | | | | Sample Count | 0 |
| | | | | Trigger | Time Step |
| | | | | Enable sliding sample window | false |
| | | | | Title | Sum of TAWSS |
| | | | | Field Function | TAWSS |
| | | `-1 Time-Step Frequency | Time-step Frequency | 1 |
| | | | Start Time-step | 0 |
| | | | Enable Stop | false |
| | | | Stop Time-step | 0 |
| | +-4 **Field Sum 2** | Parts | [Region, Region: Intersect.PartSurface\_Intersect\_Subtract, Region: Intersect.PartSurface\_Intersect\_Subtract [Intersect/Subtract], Region: Intersect.wall\_cyl, Region: Subtract.inlet, Region: Subtract.outletBig, Region: Subtract.outletSmall, Region: Subtract.PartSurface\_Subtract\_Intersect, Region: Subtract.PartSurface\_Subtract\_Intersect [Intersect/Subtract], Region: Subtract.wall] |
| | | | | Enabled | true |
| | | | | Sample Count | 0 |
| | | | | Trigger | Time Step |
| | | | | Enable sliding sample window | false |
| | | | | Title | Sum of d\_OSI |
| | | | | Field Function | d\_OSI |
| | | `-1 Time-Step Frequency | Time-step Frequency | 1 |
| | | | Start Time-step | 0 |
| | | | Enable Stop | false |
| | | | Stop Time-step | 0 |
| | +-5 **Iteration** | Maximum Plot Samples | 5000 |
| | +-6 **Max\_Local\_Re Monitor** | Report | Max\_Local\_Re |
| | | | | Enabled | true |
| | | | | Trigger | Iteration |
| | | | | Normalization Option | Off |
| | | | | Maximum Plot Samples | 5000 |
| | | `-1 Iteration Frequency | Iteration Frequency | 1 |
| | | | Start Iteration | 0 |
| | | | Enable Stop | false |
| | | | Stop Iteration | 0 |
| | +-7 **Max\_Local\_Re Monitor 2** | Report | Max\_Local\_Re |
| | | | | Enabled | true |
| | | | | Trigger | Time Step |
| | | | | Normalization Option | Off |
| | | | | Maximum Plot Samples | 5000 |
| | | `-1 Time-Step Frequency | Time-step Frequency | 1 |
| | | | Start Time-step | 0 |
| | | | Enable Stop | false |
| | | | Stop Time-step | 0 |
| | +-8 **Max\_Vel\_iteration Monitor** | Report | Max\_Vel\_iteration |
| | | | | Enabled | true |
| | | | | Trigger | Iteration |
| | | | | Normalization Option | Off |
| | | | | Maximum Plot Samples | 5000 |
| | | `-1 Iteration Frequency | Iteration Frequency | 1 |
| | | | Start Iteration | 0 |
| | | | Enable Stop | false |
| | | | Stop Iteration | 0 |
| | +-9 **Maximum 1 Monitor** | Report | Maximum 1 |
| | | | | Enabled | true |
| | | | | Trigger | Time Step |
| | | | | Normalization Option | Off |
| | | | | Maximum Plot Samples | 5000 |
| | | `-1 Time-Step Frequency | Time-step Frequency | 1 |
| | | | Start Time-step | 0 |
| | | | Enable Stop | false |
| | | | Stop Time-step | 0 |
| | +-10 **Physical Time** | Maximum Plot Samples | 5000 |
| +-18 Representations |  |  |
| | +-1 **Geometry** |  |  |
| | `-2 **Initial Surface** | Faces | 289322 |
| |   | | Edges | 1557 |
| |   `-1 Regions |  |  |
| |     `-1 Region | Faces | 289322 |
| |       | | Edges | 1557 |
| |       +-1 Boundaries |  |  |
| |       | +-1 Intersect.PartSurface\_Intersect\_Subtract | Faces | 491 |
| |       | +-2 Intersect.PartSurface\_Intersect\_Subtract [Intersect/Subtract] | Faces | 0 |
| |       | +-3 Intersect.wall\_cyl | Faces | 1685 |
| |       | +-4 Subtract.inlet | Faces | 382 |
| |       | +-5 Subtract.outletBig | Faces | 190 |
| |       | +-6 Subtract.outletSmall | Faces | 189 |
| |       | +-7 Subtract.PartSurface\_Subtract\_Intersect | Faces | 491 |
| |       | +-8 Subtract.PartSurface\_Subtract\_Intersect [Intersect/Subtract] | Faces | 0 |
| |       | `-9 Subtract.wall | Faces | 285894 |
| |       `-2 Feature Curves |  |  |
| |         `-1 Feature Curve | Edges | 1557 |
| +-19 Units | Preferred System | Systeme Internationale |
| +-20 Coordinate Systems |  |  |
| | `-1 **Laboratory** |  |  |
| |   `-1 Local Coordinate Systems |  |  |
| |     `-1 **Cartesian 1** | X Axis Direction | [-0.8675708712272394, 0.08514856688983208, 0.48996990209054336] |
| |       | | Y Axis Direction | [0.4876269705177265, -0.04785856849428662, 0.8717393504054879] |
| |       | | Z Axis Direction | [0.09767661450783963, 0.9952182067156357, -3.7752138054569784E-8] |
| |       | | Origin | [0.011386048524037028, -0.004852573164343793, 9.028042617578312E-7] m,m,m |
| |       | | Reference System | Laboratory |
| |       `-1 Local Coordinate Systems |  |  |
| +-21 Tables | Tables | 1 |
| | `-1 **velm** | Extracted | [] |
| | | Path | ..\Vmean\vmean.csv |
| +-22 Field Functions |  |  |
| | +-1 **d\_OSI** | Function Name | d\_OSI |
| | | | Inverse Distance Weight | false |
| | | | Type | Scalar |
| | | | Assembly code | (/ (\* ${WSS} ${TimeStep}) 2.49) |
| | | | Definition | ($WSS\*$TimeStep)/2.49 |
| | | | Ignore Boundary Values | false |
| | +-2 **Local\_CFL** | Function Name | Local\_CFL |
| | | | Inverse Distance Weight | false |
| | | | Type | Scalar |
| | | | Assembly code | (/ (\* (mag $${Velocity}) ${TimeStep}) (pow ${Volume} 0.333333)) |
| | | | Definition | mag($${Velocity})\* ${TimeStep}/ pow($Volume,1/3) |
| | | | Ignore Boundary Values | false |
| | +-3 **Local\_Reynolds** | Function Name | Local\_Reynolds |
| | | | Inverse Distance Weight | false |
| | | | Type | Scalar |
| | | | Assembly code | (/ (\* (\* (mag $${Velocity}) ${Ref\_length\_cells}) ${Density}) ${DynamicViscosity}) |
| | | | Definition | mag($$Velocity)\* $Ref\_length\_cells\*$Density/$DynamicViscosity |
| | | | Ignore Boundary Values | false |
| | +-4 **LocalRefinement** | Function Name | Local\_refinement |
| | | | Inverse Distance Weight | false |
| | | | Type | Scalar |
| | | | Assembly code | (if (> ${Local\_Reynolds} 10) (/ (pow ${Volume} 0.333333) 2) 0) |
| | | | Definition | ($Local\_Reynolds>10) ? pow($Volume,1/3)/ 2 : 0 |
| | | | Ignore Boundary Values | false |
| | +-5 **Ones** | Function Name | Ones |
| | | | Inverse Distance Weight | false |
| | | | Type | Scalar |
| | | | Assembly code | 1 |
| | | | Definition | 1 |
| | | | Ignore Boundary Values | false |
| | +-6 **OSI** | Function Name | OSI |
| | | | Inverse Distance Weight | false |
| | | | Type | Scalar |
| | | | Assembly code | (\* 0.5 (- ${Ones} (/ (abs ${d\_OSI}) ${TAWSS}))) |
| | | | Definition | 0.5\*($Ones-abs($d\_OSI)/$TAWSS) |
| | | | Ignore Boundary Values | false |
| | +-7 **Ref\_length\_cells** | Function Name | Ref\_length\_cells |
| | | | Inverse Distance Weight | false |
| | | | Type | Scalar |
| | | | Assembly code | (pow ${Volume} 0.333333) |
| | | | Definition | pow($Volume,1/3) |
| | | | Ignore Boundary Values | false |
| | +-8 **RRT** | Function Name | RRT |
| | | | Inverse Distance Weight | false |
| | | | Type | Scalar |
| | | | Assembly code | (/ 2.49 (abs ${d\_OSI})) |
| | | | Definition | 2.49/abs($d\_OSI) |
| | | | Ignore Boundary Values | false |
| | +-9 **sigmaW** | Function Name | sigmaW |
| | | | Inverse Distance Weight | false |
| | | | Type | Vector |
| | | | Assembly code | (\* (dot $${WallShearStress} (unit $${Area})) (unit $${Area})) |
| | | | Definition | dot($$WallShearStress,unit($$Area))\*unit($$Area) |
| | | | Ignore Boundary Values | false |
| | +-10 **TAWSS** | Function Name | TAWSS |
| | | | Inverse Distance Weight | false |
| | | | Type | Scalar |
| | | | Assembly code | (/ (abs (\* ${WSS} ${TimeStep})) 2.49) |
| | | | Definition | abs($WSS\*$TimeStep)/2.49 |
| | | | Ignore Boundary Values | false |
| | +-11 **tauWS** | Function Name | tauWS |
| | | | Inverse Distance Weight | false |
| | | | Type | Vector |
| | | | Assembly code | (- $${WallShearStress} $${sigmaW}) |
| | | | Definition | $$WallShearStress - $$sigmaW |
| | | | Ignore Boundary Values | false |
| | +-12 **WSS** | Function Name | WSS |
| | | | Inverse Distance Weight | false |
| | | | Type | Scalar |
| | | | Assembly code | (mag $${tauWS}) |
| | | | Definition | mag($$tauWS) |
| | | | Ignore Boundary Values | false |
| +-23 Volume Shapes |  |  |
| +-24 Update Events | Event Count | 0 |
| | | Event Names |  |
| +-25 Data Set Functions | Data Directory | function\_data |
| +-26 User Code |  |  |
| +-27 Layouts |  |  |
| | `-1 default |  |  |
| +-28 Data Mappers |  |  |
| +-29 Motions |  |  |
| | `-1 **Stationary** |  |  |
| +-30 Reference Frames |  |  |
| | `-1 **Lab Reference Frame** |
